# Medical Procedures and Risk for Sporadic Creutzfeldt-Jakob Disease, Japan, 1999–2008

**DOI:** 10.3201/eid1502.080749

**Published:** 2009-02

**Authors:** Tsuyoshi Hamaguchi, Moeko Noguchi-Shinohara, Ichiro Nozaki, Yosikazu Nakamura, Takeshi Sato, Tetsuyuki Kitamoto, Hidehiro Mizusawa, Masahito Yamada

**Affiliations:** Kanazawa University Graduate School of Medical Science, Kanazawa, Japan (T. Hamaguchi, M. Noguchi-Shinohara, I. Nozaki, M. Yamada); Jichi Medical University, Shimotsuke, Japan (Y. Nakamura); Kohnodai Hospital, Ichikawa, Japan (T. Sato); Tohoku University Graduate School of Medicine, Sendai, Japan (T. Kitamoto); Tokyo Medical and Dental University, Tokyo, Japan (H. Mizusawa); Creutzfeldt-Jakob Disease Surveillance Committee, Japan (Y. Nakamura, T. Sato, J. Mizusawa, T. Kitamoto, M. Yamada)

**Keywords:** Prion, Creutzfeldt-Jakob disease, medical procedure, surgery, neurosurgery, ophthalmic surgery, blood transfusion, Japan, research

## Abstract

To elucidate the association between medical procedures and sporadic Creutzfeldt-Jakob disease (sCJD), we analyzed medical procedures (any surgical procedure, neurosurgery, ophthalmic surgery, and blood transfusion) for patients registered by the CJD Surveillance Committee in Japan during 1999–2008. We conducted an age-stratified case–control study with 753 sCJD patients and 210 controls and a study of patients who underwent neurosurgical or ophthalmic surgical procedures at the same hospital. Although the control group was relatively small, no evidence was found that prion disease was transmitted through the investigated medical procedures before onset of sCJD. After onset of sCJD, 4.5% of the sCJD patients underwent operations, including neurosurgical for 0.8% and ophthalmic for 1.9%; no special precautions against transmission of prion diseases were taken. Fortunately, we have not identified patients with prion disease attributed to these operations. Our findings indicate that surgical procedures or blood transfusion had little effect on the incidence of sCJD.

## Abstract 2

Surgery or blood transfusion had little effect on the incidence of sCJD.

Prion disease is characterized by spongiform change and abnormal prion protein deposition in the brain and is transmissible under certain situations. Human prion disease is divided into 3 categories: genetic prion diseases with mutations of the prion protein (PrP) gene; prion diseases acquired by transmission of the prion through exposure to contaminated materials, including iatrogenic transmission; and sporadic Creutzfeldt-Jakob disease (sCJD) with no PrP mutation or evidence of exposure to prion. To date, >400 patients with iatrogenic CJD, who received prions through contaminated neurosurgical instruments, intracerebral electroencephalographic electrodes, human pituitary hormone, corneal transplants, or dura mater grafts, have been reported (*1*). Furthermore, some case–control studies reported that medical procedures were possible risk factors for sCJD (*2**–**6*). However, other studies did not demonstrate any significant association between medical procedures and sCJD (*7**–**10*).

After a results of a case–control study that found an association between CJD and medical procedures was reported from Japan in 1982 (*2*), 132 patients with dura mater graft–associated CJD (dCJD) have been found in Japan (*11**,**12*); however, no recent studies have investigated medical procedures as a risk for acquiring sCJD. In Japan, 66 (8.6%) of 766 patients with prion diseases had iatrogenic cases that were all dCJD (*12*), and the outbreak of iatrogenic CJD required a new study about the association between sCJD and medical procedures in Japan. Here we analyzed the role of medical procedures in cases of sCJD by using relevant data from CJD surveillance in Japan.

## Methods

### Patients

We investigated 1,339 patients with suspected prion diseases who had been registered by the CJD Surveillance Committee in Japan from April 1999 through February 2008. The surveillance system was initiated in April 1999, and each patient was prospectively assessed with a surveillance protocol that assembled information about life history; previous medical history, including the history of surgical treatment and blood transfusion; clinical history; laboratory data; and results of molecular genetic and pathologic examinations. Information on patients with suspected prion diseases were obtained through 1) the application for registration with the Japanese Intractable Diseases Information Center (www.nanbyou.or.jp/english/nan_kenkyu_45.htm) by each patient’s family, 2) the law on infectious diseases, or 3) request for genetic or cerebrospinal fluid analyses sent to members of the CJD Surveillance Committee by the physicians. In Japan, 123 diseases have been defined as intractable disease, and for 45 of them, including prion diseases, patients receive additional economic support for medical costs. Furthermore, medical doctors must report patients suspected of having prion disease to the local public health department within 7 days after the diagnosis, according to the law on infectious diseases (which has been enforced since April 1999 in Japan to monitor some specific infectious diseases). After written consent approved by the Institutional Ethics Committee was obtained from each patient’s family, members of the CJD Surveillance Committee directly examined the patient and collected data from the clinical records. For each patient with a history of surgery, we collected information about the underlying disease from the patient’s family, including the date and hospital in which the operation was performed. For each patient with a history of blood transfusion, we collected information about the date of blood transfusion. Most information was collected by interviewing the patient’s family members.

On the basis of discussions by the CJD Surveillance Committee, we confirmed or denied the diagnosis of prion disease in each case. In patients with a confirmed diagnosis of prion disease, we classified prion diseases into 4 categories: sCJD, acquired prion disease, genetic prion disease, and unclassified prion disease. sCJD was diagnosed according to the revised classical criteria established by Masters et al. (*13*): definite CJD (neuropathologically confirmed spongiform encephalopathy or abnormal prion protein deposition in the brain); and probable CJD (neuropathologically unconfirmed cases showing progressive dementia, periodic sharp-wave complexes on electroencephalogram, and at least 2 of the following features: myoclonus, pyramidal signs/extrapyramidal signs, cerebellar signs or visual symptoms, and akinetic mutism). Acquired prion diseases included iatrogenic CJD, in which the criteria for sCJD were applied for a diagnosis with a history of iatrogenic exposure, and variant CJD, in which the diagnosis was based on the World Health Organization (WHO) 2001 criteria (*14*). Regarding the accuracy of the diagnosis of genetic prion diseases, pathologically verified cases were defined as “definite,” and cases demonstrating mutations in the PrP gene and neuropsychiatric manifestations compatible with prion diseases were defined as “probable.” We selected patients with definite or probable sCJD for analysis.

Patients who did not receive a diagnosis of prion diseases were classified into 3 categories: prion diseases definitely denied; prion diseases probably denied; and diagnosis unclear. “Prion diseases definitely denied” indicated patients whose conditions were definitively diagnosed as diseases other than prion diseases, and “prion diseases probably denied” indicated patients for whom the diagnosis of prion diseases was clearly unlikely due to the improving or nonprogressive disease course or for other reasons, although a definitive diagnosis of another disease was not established. Because patients with “prion diseases definitely denied” or “prion disease probably denied” had no or little possibility of prion disease, we selected these cases as the controls in our case–control study.

### Surgical Procedures and Blood Transfusions before Onset of sCJD

To estimate the risk for sCJD through past surgery or blood transfusion, we performed a case–control study. Operations were divided into the following categories: neurosurgery, ophthalmic surgery, and surgery other than neurosurgery or ophthalmic surgery (other surgery), because neurosurgery or ophthalmic surgery for those with prion diseases are categorized in the guidelines of the CJD Incident Panel in the United Kingdom as high- or medium-risk procedures for transmission of infective PrP (*15*). In these guidelines, procedures involving the olfactory epithelium are also categorized as medium risk (*15*). However, the number of persons who underwent the operation possibly involving the olfactory epithelium is too small to be estimated by statistical analysis (2 sCJD patients and 2 controls underwent surgery for sinusitis), and we categorized these operations as other surgery. Neurosurgery included operations on the brain, cerebral blood vessels, and spinal cord. Ophthalmic surgery included all operations involving the eyeball and optic nerve. Other surgery included all surgical procedures other than neurosurgery and ophthalmic surgery. Furthermore, the committee performed a detailed investigation of sCJD patients who underwent neurosurgery or ophthalmic surgery at a hospital where other patients with any type of prion disease had ever undergone neurosurgery or ophthalmic surgery.

### Surgical Procedures after Onset of sCJD

We analyzed sCJD patients who underwent surgical procedures after the onset of sCJD because such procedures might cause secondary transmission of the disease through contaminated instruments. In particular, for neurosurgery and ophthalmic surgery, we investigated the reason for the operation, interval between the operation and onset of sCJD symptoms, age at onset of sCJD, and symptoms at onset of sCJD.

### Statistical Analyses

Between the sCJD and control groups, age at onset was compared by Student *t* test, and medical procedures before the onset of diseases were compared by Fisher exact test. The case–control study of surgical procedures and blood transfusions before the onset of diseases was estimated by logistic-regression analysis. Because age at onset was different among sCJD patients (mean ± SD, 67.7 ± 9.5 years) and controls (59.3 ± 16.6 years) (p<0.0001), we divided the sCJD patients and controls into 3 categories according to age at disease onset; 31–50 years, 51–70 years, and >71 years. We performed a single regression analysis for any operation, neurosurgical procedure, ophthalmic surgical procedure, other operation, and blood transfusion in each age group. The strength of association between sCJD and putative risk factors was assessed by the odds ratios and 95% confidence intervals. Significance was defined as p<0.05. Statistical analyses were performed by using StatView J-7.5 (Abacus Concepts, Berkeley, CA, USA).

## Results

A total of 990 patients received a diagnosis of definite or probable prion disease. Summary of the characteristics of patients with prion diseases is shown in Table 1, in which 760 patients with sCJD are included. There were 221 patients with “prion disease definitely denied” and “prion disease probably denied.” Seven sCJD patients and 11 control patients were excluded from the case–control study because information on medical history was not sufficient for analysis. Diagnoses of the 210 control patients is shown in Table 2.

**Table 1:** Characteristics of patients with definite or probable prion disease, Japan, 1999–2008*

Type of prion disease	No. (%) patients
Sporadic CJD	760 (76.8)
Genetic prion diseases	167 (16.9)
Acquired prion diseases†	62 (6.3)
Unclassified CJD	1 (0.1)
Total	990

*CJD, Creutzfeldt-Jakob disease. †Acquired prion diseases included 61 cases of dura mater CJD and 1 case of variant CJD.

**Table 2:** Diagnoses for 210 controls in case–control study of sCJD, Japan, 1999–2008*

Disease	No. diagnoses
Encephalitis	27
Alzheimer disease	21
Frontotemporal dementia	15
Metabolic encephalopathy	15
Cerebrovascular disorders	12
Spinocerebellar degeneration	12
Corticobasal degeneration	9
Epilepsy	7
Psychiatric disorders	7
Hypoxic encephalopathy	7
Hashimoto encephalopathy	6
Dementia with Lewy bodies	6
Paraneoplastic syndrome	5
Mitochondrial encephalopathy	4
Malignant lymphoma	3
Other disorders	54

*sCJD, sporadic Creutzfeldt-Jakob disease.

### Medical Procedures before Onset of sCJD

Frequencies of medical procedures before the onset of sCJD in sCJD patients and in controls are compared in Table 3. For both the sCJD and control groups, ≈50% had a history of surgery, and ≈10% had received a blood transfusion. No significant differences were found between them in frequency of any surgery, neurosurgery, ophthalmic surgery, other surgery, or blood transfusion (Table 3). In the logistic-regression analysis, no significant risk was associated with any medical procedures investigated in this study (Table 4).

**Table 3:** Medical procedures before disease onset, case–control study of sCJD, Japan, 1999–2008*

Medical procedures	sCJD case-patients, no. (%), n = 753	Controls, no. (%), n = 210
Surgery	372 (49.4)	104 (49.5)
Neurologic	25 (3.3)	13 (6.2)
Ophthalmic	42 (5.6)	11 (5.2)
Other	337 (44.8)	89 (42.4)
Blood transfusion	78 (10.4)	20 (9.5)

*sCJD, sporadic Creutzfeldt-Jakob disease. p values were not significant.

**Table 4:** Medical procedures and risk for sCJD, by age at disease onset, Japan, 1999–2008*

Age range, y	Data category	Total no. patients	Any surgery	Neurosurgery	Ophthalmic surgery	Other surgery	Blood transfusion
31–50	sCJD	32	50.0%	6.3%	6.3%	40.6%	3.1%
	Control	37	45.9%	10.8%	2.7%	37.8%	5.4%
	OR		1.66	0.38	2.15	0.78	0.64
	95% CI		0.04–74.09	0.02–6.64	0.05–101.51	0.02 – 33.39	0.05–9.09
	p value		0.79	0.50	0.70	0.90	0.74
51–70	sCJD	414	43.7%	1.7%	2.2%	41.8%	9.4%
	Control	97	46.4%	5.2%	3.1%	40.2%	11.3%
	OR		0.18	0.69	2.71	5.57	0.84
	95% CI		0.02–1.73	0.13–3.62	0.24–30.38	0.62–50.05	0.40–1.77
	p value		0.14	0.66	0.42	0.13	0.64
>71	sCJD	317	57.0%	5.2%	10.1%	49.2%	12.4%
	Control	60	65.0%	6.7%	10.0%	56.7%	11.7%
	OR		0.81	0.76	1.15	0.83	1.27
	95% CI		0.15–4.37	0.15–3.80	0.38–3.48	0.17–4.02	0.52–3.10
	p value		0.80	0.74	0.81	0.82	0.60

*sCJD, sporadic Creutzfeldt-Jakob disease; OR, odds ratio; CI, confidence interval.

Five sCJD patients had a history of neurosurgery or ophthalmic surgery at hospitals where neurosurgery or ophthalmic surgery had been performed on patients in whom prion disease later developed (Table 5); intervals between operations at the same hospitals were >3 years (Table 5).

**Table 5:** Characteristics of 5 sCJD patients who underwent neurosurgery or ophthalmic surgery at hospitals where other patients with prion diseases had previously undergone neurosurgery or ophthalmic surgery, Japan, 1999–2008*

Patient	Type of CJD	Onset of CJD	Date of surgery	Reason for surgery
1	sCJD	2003 Aug	1991 Aug	Subarachinoid hemorrhage
	dCJD	2001 May	1976	Spinal cord tumor
			1986 Aug	Spinal cord tumor
2	sCJD	2002 Feb	1994 Sep	Subdural hematoma
			1997 Sep	Cataract
	dCJD	1998 Jan	1987 Jan	Meningioma
3	sCJD	2001 Jan	1989 Apr	Subarachinoid hemorrhage
	dCJD	1995 Jul	1980 Jul	Aneurysm
4	sCJD	2001 Jul	1999	Spinal cord lesion (details unknown)
	dCJD	2001 Aug	1978 Sep	Astrocytoma
5	sCJD	2002 May	2002 Apr	Cataract
	sCJD	2002 May	1997 Aug	Cataract
			1999 Jan	Cataract

*CJD, Creutzfeldt-Jakob disease; sCJD, sporadic CJD; dCJD, Creutzfeldt-Jakob disease associated with cadaveric dura mater graft.

### Surgical Procedures after Onset of sCJD

Except for 2 patients suspected of having prion disease, who had undergone brain biopsy with disposable instruments, 34 (4.5%) of 760 sCJD patients underwent some type of surgical procedure before the diagnosis of prion disease, including neurosurgery in 6 (0.8%), ophthalmic surgery in 14 (1.8%), and other surgery in 16 (2.1%). The 6 case-patients who underwent neurosurgery had these operations within 3 months after sCJD onset: procedures performed for subdural hematoma (n = 3), aneurysm (n = 2), and menigioma (n = 1) (Table 6). All 14 case-patients who underwent ophthalmic surgery underwent operations for cataracts, and 7 of these patients had had visual disturbance as an initial symptom of sCJD (Table 7). Among 5 patients for whom information on the effects of ophthalmic surgery could be obtained, 2 had some improvement of visual symptoms after surgery, but the other 3 patients had no improvement. Although both cataracts and sCJD could contribute to the visual symptoms, sCJD would contribute to visual symptoms in patients who had no effects of ophthalmic surgery. We have obtained information about instrument cleaning and sterilization procedures for 3 of 5 patients who underwent neurosurgery and for 5 of 14 patients who underwent ophthalmic surgery after the onset of sCJD. All surgeons reused some of the surgical instruments, but according to the WHO guidelines (*16*), the sterilization methods of the instruments were not appropriate for eliminating infectious PrP, including the use of ethylene oxide gas or incomplete autoclaving.

**Table 6:** Data for sCJD patients who underwent neurosurgery after onset of sCJD symptoms, Japan, 1999–2008*

Patient no.	Reason for surgery	Interval between onset of sCJD symptoms and surgery, mo	Age at onset of sCJD, y	Symptom at onset of sCJD
1	Subdural hematoma	0	71	Dementia
2	Subdural hematoma	0	77	Apathy
3	Subdural hematoma	1	57	Dementia
4	Meningioma	1	74	Vertigo
5	Aneurysm	2	46	Dementia
6	Aneurysm	3	67	Vertigo

*sCJD, sporadic Creutzfeldt-Jakob disease.

**Table 7:** Data for sCJD patients who had ophthalmic surgery for cataracts after onset of sCJD symptoms, Japan, 1999–2008*

Patient no.	Interval between onset of sCJD symptoms and surgery, mo	Age at onset of sCJD, y	Symptom at onset of sCJD
1	0	60	Gait disturbance
2	0	61	Dementia
3	0	63	Visual impairment
4	0	71	Visual impairment
5	0	74	Visual impairment
6	0	74	Visual impairment
7	1	66	Dementia
8	1	74	Depression
9	1	85	Visual impairment
10	2	79	Tremor
11	4	81	Visual impairment
12	8	77	Anxiety
13	10	57	Dementia
14	14	64	Visual impairment

*sCJD, sporadic Creutzfeldt-Jakob disease.

## Discussion

In this case–control study, we found no evidence of increased sCJD risk associated with patient’s history of surgical procedures or blood transfusions. In the previous case–control study and in our study, receipt of a blood transfusion was not shown to be a significant risk for CJD (*2**–**10*). However, whether surgical procedures contribute to the risk for sCJD has been controversial. Our results, in which any operation was not a significant risk for sCJD, were consistent with results of 2 previous large case–control studies (*8**,**9*) and a reanalysis of results of 3 case–control studies (*10*). Even in the studies with positive results, some different results were provided when the surgical procedures were categorized by affected organ. One previous case–control study indicated significant risk for sCJD after neurosurgical procedures (*3*), but no significant risk was shown in other studies (*5**,**6**,**8**–**10*). Ophthalmic surgery was reported as causing significant risk for sCJD in a case–control study in Australia (*4*) but not in other studies (*5**,**6**–**10*). In a recent study in the United Kingdom (*6*), the increased risk associated with having undergone surgical procedures was restricted to the category “other surgery,” which included such procedures as sutures to skin, and the association largely disappeared when the whole of the other-surgery category was excluded. These different results may show little possibility for transmission of infectious PrP through surgical procedures, although we cannot exclude the possibility that such transmission occurs occasionally because iatrogenic CJD exists.

The conflicting results in case–control studies, including ours, may be explained by differences in the area, race, period in which studies were performed, number of patients, and methods as discussed below. Our study, which attempted to determine when medical procedures were associated with an increased risk for sCJD, had the largest number of sCJD patients in case–control studies to date. The relatively small number of controls is a potential limitation. In case–control studies, methods of obtaining data from controls should be the same as those from patients. In our study, patients in the groups “prion diseases definitely denied” or “prion diseases probably denied” in our CJD surveillance, who had no or little possibility of having prion disease, were used as the controls. Therefore, data from controls could be collected at the same level of precision as those from the sCJD cases. Because the ages of the sCJD patients and controls were significantly different, age-stratified analysis was required in our study. A recent study reported that some methodologic differences might partially explain conflicting data regarding the association between surgical procedures and CJD (*17*). The report suggested that the use of controls from the community would be preferable to using those from the hospital because community-based controls are often more representative and would result in a more valid comparison (*17*). Furthermore, using proxy informants for controls may be advisable for the purpose of comparability with case-patients, although this practice does not necessarily offset biases in data ascertainment (*17*). In our case–control study, we used proxy informants for controls who were recruited from hospitals under the same condition as the sCJD case-patients.

Regarding the 5 sCJD patients with a history of neurosurgical or ophthalmic surgical procedures at hospitals where other patients with prion disease had previously undergone such procedures, we consider that the possibility of transmission through these procedures was extremely limited because the intervals between procedures and the acquisition of sCJD had been >3 years for all patients. According to the Incident Panel in the United Kingdom, most instruments that have gone through 10 cycles of use and decontamination are unlikely to pose a substantial risk (*15*). We assume that all instruments had gone through >10 cycles of use during the 3-year interval, and almost no infectivity remained on the instruments. In Japan, a large number of dCJD patients have been recognized with no other types of iatrogenic CJD (*11**,**12*); this study confirmed that no surgically transmitted cases occurred among patients with sCJD.

It is noteworthy that 4.5% of the sCJD patients underwent some types of surgical procedures after the disease onset, including neurosurgical (0.8%) and ophthalmic procedures (1.8%). Through surgical instruments, neurosurgical operations may transmit high infectivity from the brain tissues of sCJD patients, and ophthalmic operations may transmit moderate infectivity of the eye tissues in cases of cataracts (*15*). In this study, all these neurosurgical and ophthalmic procedures were performed without suspicion of prion diseases or special precautions to reduce the risk for secondary transmission of prion infection through the instruments. These findings suggest that delayed diagnosis of sCJD would be linked to increased risk for secondary transmission of prion diseases through surgical instruments. In neurosurgical procedures, the symptoms of sCJD were misdiagnosed as those of other neurologic diseases, and operations were performed near the time of disease onset. In terms of ophthalmic surgery, all patients underwent operations for cataracts, and 7 (50%) of 14 patients had visual disturbances as an initial symptom of sCJD. These data are similar to those in a report from the United Kingdom (*18*). Visual disturbances might prompt ophthalmic surgery. More seriously, 3 patients underwent operations >8 months after sCJD onset. In this study, all surgeons who provided information reused the surgical instruments with incomplete sterilization, and the potential for infection was the same as in our previous study of ophthalmic surgery (*19*).

Neurosurgeons and ophthalmologists should become better informed about prion diseases and the necessity of using disposable instruments whenever possible. Furthermore, a more sensitive method for early diagnosis of sCJD is needed because clinical diagnosis is sometimes difficult, particularly in atypical sCJD cases, such as MM2, MV2, VV1, or VV2 types (*20**–**23*), according to 6 phenotypes of sCJD divided by codon 129 polymorphisms of PrP (methionine/valine) and type of infectious PrP by Western blotting (*24*). Even neurologists may misdiagnose the initial stage of the atypical sCJD cases as being another neurodegenrative disease such as Alzheimer disease and progressive supranuclear palsy (*20*). Moreover, patients who have undergone surgical procedures with possibly contaminated instruments need to undergo a risk assessment with long-term follow-up after careful ethical consideration. Since June 2004, we have identified and monitored all patients who underwent neurosurgical procedures with possibly contaminated instruments, CJD has developed in none of those patients.

In conclusion, we did not demonstrate any evidence of increased risk for sCJD associated with a history of surgery or blood transfusion in the Japanese surveillance system. However, the fact that some patients had surgeries, including neurosurgery, even after the onset of sCJD indicates that we cannot deny any possibility of transmission of prion diseases by medical procedures. Neurosurgeons, ophthalmologists, and other surgeons need to focus more attention on prion diseases to reduce the iatrogenic risk, as well as realize that prolonged, careful surveillance of prion diseases is necessary.
